# Increased cerebrospinal fluid fibrinogen in major depressive disorder

**DOI:** 10.1038/srep11412

**Published:** 2015-06-17

**Authors:** Kotaro Hattori, Miho Ota, Daimei Sasayama, Sumiko Yoshida, Ryo Matsumura, Tomoko Miyakawa, Yuuki Yokota, Shinobu Yamaguchi, Takamasa Noda, Toshiya Teraishi, Hiroaki Hori, Teruhiko Higuchi, Shinichi Kohsaka, Yu-ichi Goto, Hiroshi Kunugi

**Affiliations:** 1 Department of Mental Disorder Research, National Institute of Neuroscience, National Center of Neurology and Psychiatry, Tokyo 187-8502, Japan; 2 Translational Medical Center, National Center of Neurology and Psychiatry, Tokyo 187-8551, Japan; 3 National Center of Neurology and Psychiatry Hospital, Tokyo 187-8551, Japan; 4 National Center of Neurology and Psychiatry, Tokyo 187-8551, Japan; 5 National Institute of Neuroscience, National Center of Neurology and Psychiatry, Tokyo 187-8502, Japan

## Abstract

Major depressive disorder (MDD) presumably includes heterogeneous subgroups with differing pathologies. To obtain a marker reflecting such a subgroup, we analyzed the cerebrospinal fluid (CSF) levels of fibrinogen, which has been reported to be elevated in the plasma of patients with MDD. Three fibrinogen-related proteins were measured using aptamer-based analyses and CSF samples of 30 patients with MDD and 30 controls. The numbers of patients with an excessively high level (>99 percentile of the controls) was significantly increased (17 to 23%). Measurement reproducibility of these results was confirmed by an ELISA for fibrinogen (Pearson’s r = 0.77). In an independent sample set from 36 patients and 30 controls, using the ELISA, results were similar (22%). When these two sample sets were combined, the number of patients with a high fibrinogen level was significantly increased (15/66; odds ratio 8.53; 95% confidence interval 1.9–39.1, p = 0.0011). By using diffusion tensor imaging, we found white matter tracts abnormalities in patients with a high fibrinogen level but not those patients with a normal fibrinogen level, compared with controls. Plasma fibrinogen levels were similar among the diagnostic groups. Our results point to a subgroup of MDD represented by increased CSF fibrinogen and white matter tract abnormalities.

Major depressive disorder (MDD) is supposed to include heterogeneous subgroups with different biological mechanisms contributing to their disease^1^. Meta-analyses have concluded that some molecules differ significantly between patients with MDD and controls: blood brain-derived neurotrophic factor^2^, proinflammatory cytokines^3^,^4^,^5^, plasma tryptophan^6^, and stress hormone responses^7^, to name a few. We have also obtained evidence for such markers in our samples^6^,^8^. These markers may be useful; however, no biomarker has yet been established for the diagnosis or subtyping of MDD in the daily clinical practice.

Cerebrospinal fluid (CSF) is derived mainly from capillaries in the brain tissue, rather than the choroid plexus, and is in continuity with the brain interstitial fluid^9^. Therefore, molecules released from brain cells can directly diffuse into the CSF. There are established CSF biomarkers for brain disorders, such as tau, phosphorylated tau, and β-amyloid proteins for Alzheimer’s disease (AD), which can be used to diagnose AD with a high (~90%) sensitivity and specificity^10^. Thus, although there are some disadvantages, including the risk of headache, CSF is likely to reflect brain pathology in certain cases, and using CSF samples may be a good way to search for biomarkers of various brain disorders. Indeed, we previously found that interleukin (IL)-6 levels were increased in the CSF of patients with MDD and schizophrenia^11^.

Fibrinogen is crucial for blood clotting, inflammation, and angiogenesis. Previous studies showed that plasma fibrinogen levels were elevated in patients with MDD^12^,^13^,^14^,^15^,^16^. However, to our knowledge, no study has examined CSF fibrinogen levels in patients with MDD. We therefore examined fibrinogen levels in two independent case-control samples and explored the possible relationship between fibrinogen and brain structures assessed with magnetic resonance imaging (MRI).

## Results

### Measurements of fibrinogen related proteins by SOMAscan

**Figure 1:**
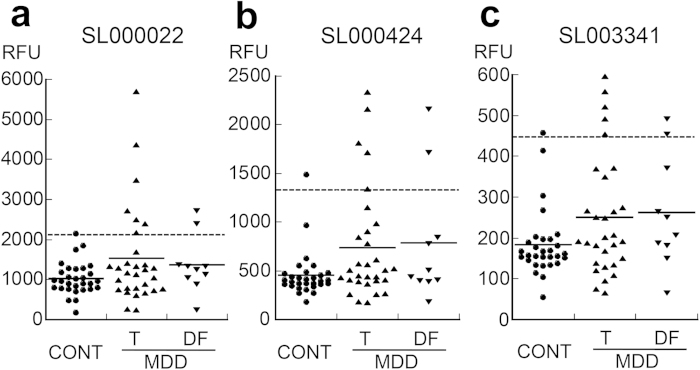
SOMAscan measurements of three fibrinogen-related proteins. SL000022 (**a**), SL000424 (**b**), and SL003341 (**c**), in CSF samples from patients with MDD (n = 30, including 10 drug-free cases) and controls (n = 30). Plain and dotted lines indicate the mean in each group and 99 percentile of the controls, respectively. CSF, cerebrospinal fluid; RFU, relative fluorescent unit; CONT, control; MDD, major depressive disorder; T, total patients with MDD; DF, drug-free patients with MDD.

We evaluated the levels of three fibrinogen-related molecules in SOMAscan analyses: SL000022, SL000424, and SL003341 which were supposed to represent the fibrin D-dimer, fibrinogen, and fibrinogen γ-chain, respectively, in the first sample. Seven patients with MDD had an abnormally high level of SL000022 (>2066 RFU, 99 percentile of the controls) (Fig. 1a). Two of the 7 cases were drug-free patients. The number of cases with an abnormally high level was increased in patients with MDD (23% vs 3%; OR, 8.83; 95% CI, 1.01–77.0; p = 0.026). The mean SL000022 level in patients tended to be higher than that of controls (1527 ± 1216 vs 1029 ± 408, F(1, 55) = 3.65, p = 0.06, partial η^2^ = 0.06). Five patients had an abnormally high level of SL000424 (>1338 RFU, Fig. 1b), including two drug-free cases. The number of cases with an abnormally high level tended to be increased in patients (17% vs 3%; OR, 5.80; 95%CI, 0.64-53.0; p = 0.097). The mean SL000424 level in patients was significantly higher than in controls (739 ± 582 vs 456 ± 238, F(1,55) = 5.0, p = 0.03, partial η^2^ = 0.08). Five patients had an abnormally high level of SL003341 (>445 RFU, Fig. 1c), including two drug-free cases. The number of cases with abnormally high levels tended to be increased in patients with MDD (17% vs 3%; OR, 5.80; 95%CI, 0.64-53.0, p = 0.097). The mean SL003341 level in patients tended to be higher than that in controls (251 ± 148 vs 184 ± 82, F(1, 55) = 3.85, p = 0.06, partial η^2^ = 0.065).

The expression pattern of the three fibrin-related proteins correlated with each other. In particular, SL000424 and SL003341 showed a nearly perfect correlation (SL000022 and SL000424, Pearson’s r = 0.50, p = 4.2 × 10^−5^; SL000022 and SL003341, r = 0.66, p=9.3 × 10^−9^; SL000424 and SL003341, r = 0.93, p = 2.2 × 10^−27^; Supplemental Figs. S1-3).

### ELISA vs SOMAscan

**Figure 2:**
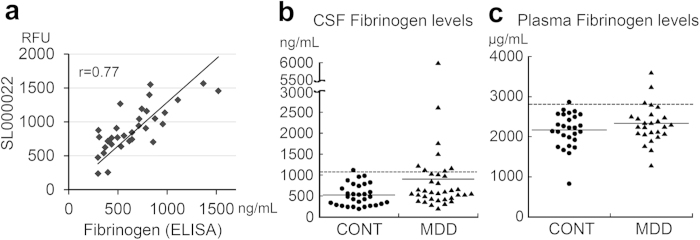
ELISA measurements of fibrinogen. (**a**) Correlation between CSF fibrinogen (ELISA) and SL000022 (SOMAscan) levels (N = 32). (**b**) CSF fibrinogen levels in the second sample. (**c**) Plasma fibrinogen levels in plasma sample. Plain and dotted lines in (**b**) and (**c**) indicate the mean in each group and 99 percentile of the controls, respectively. CSF, cerebrospinal fluid; RFU, relative fluorescent unit; r, Pearson’s correlation coefficient; ELISA, enzyme-linked immunosorbent assay; CONT, control; MDD, major depressive disorder.

The SOMAscan uses aptamers that can bind respective proteins. It was unclear as to whether it distinguished the above three proteins (Somalogic Co., Response to inquiry). To specify which molecules the three SOMAscan aptamers had measured, we examined the correlation between SOMAscan and ELISA measurements using each ELISA kit and the samples analyzed by SOMAscan (n = 32). There was a strong correlation between fibrinogen (ELISA) and SL000022 (r = 0.77, p = 4.4 × 10^−7^, Fig. 2a, Supplemental Table S1). There was a modest correlation between fibrinogen γ-chain (ELISA) and SL000022 (r = 0.40, p < 0.05, Supplemental Table S1, Supplemental Fig. S4). No clear correlation was detected between SL000022 and fibrin D-dimer, between SL000424 and three ELISAs, or between SL003341 and three ELISAs (Supplemental Table S1).

### Fibrinogen levels in second samples

We measured CSF fibrinogen using ELISA in the second sample. Here again the cutoff value of fibrinogen was defined as 99 percentile of the levels in the controls. Eight of 36 patients showed an abnormally high fibrinogen level (>1077 ng/mL, Fig. 2b). The number of subjects with an abnormally high level was increased in patients compared with controls (22% vs 3%; OR, 8.29; 95%CI 0.97–70.6; p = 0.027). The mean fibrinogen level tended to be higher in patients than in controls (904 ± 994 vs 527 ± 258, F(1, 61) = 4.00, p = 0.051, partial η^2^ = 0.062).

When the data (categorical data of patients and controls from each cutoff value) from the two sample sets were combined, the numbers of patients with a high fibrinogen level was significantly increased compared with controls (15/66 patients vs 2/60 controls; 23% vs 3%; OR, 8.53; 95%CI, 1.86–39.1; p = 0.0011). When data from the two samples sets were combined after z score transformation, the mean fibrinogen level in patients was significantly higher than in controls (1.35 ± 3.46 vs 0.0 ± 0.99, F(1,121) = 7.42, p=0.007, partial η^2^ = 0.058).

### Clinical features of subjects with high fibrinogen levels

We analyzed clinical features of patients with high fibrinogen levels in both the first and second samples. Compared with patients with a normal fibrinogen level, patients with a high fibrinogen level had a significantly higher mean age (F(1,62) = 12.5, p = 0.0008, partial η^2^ = 0.168, Table 1, Supplemental Fig. S5). The mean total HAM-D 17 score in patients with a high fibrinogen level was higher than in patients with a normal fibrinogen level, although the difference was not statistically significant (F(1, 60) = 3.19, p = 0.079, partial η^2^ = 0.05, Table 1, Supplemental Fig. S6). No significant differences were detected in the imipramine-equivalent antidepressant doses or white cell numbers in CSF. The total protein concentration was significantly higher in patients with a high fibrinogen level compared with those with a normal fibrinogen level (F(1, 59) = 42.3, p = 1.9 × 10^−8^, partial η^2^ = 0.42).

### Plasma fibrinogen levels

The fibrinogen level in plasma was ~1000 times higher than that in CSF. There was no significant correlation between plasma and CSF fibrinogen levels (r = 0.11, p = 0.32, Supplemental Fig. S7). Subjects with high plasma fibrinogen levels (>99 percentile, that is >2820 μg/mL) included 3 of 26 patients and 1 of 27 controls (Fig. 2c). The number of subjects with a high plasma fibrinogen level did not differ significantly between the two groups (12% vs 4%; OR, 3.25; 95%CI, 0.32–33.4; p = 0.31). There was no significant difference in mean plasma fibrinogen level between the patients and controls (2328 ± 478 vs 2166 ± 434, F(1,48) = 1.8, p = 0.18, partial η^2^ = 0.037). None of the four subjects (3 patients and 1 control) with a high plasma fibrinogen level had an abnormally high CSF fibrinogen level.

### Relationship between the CSF fibrinogen and cerebral white matter integrity

**Figure 3:**
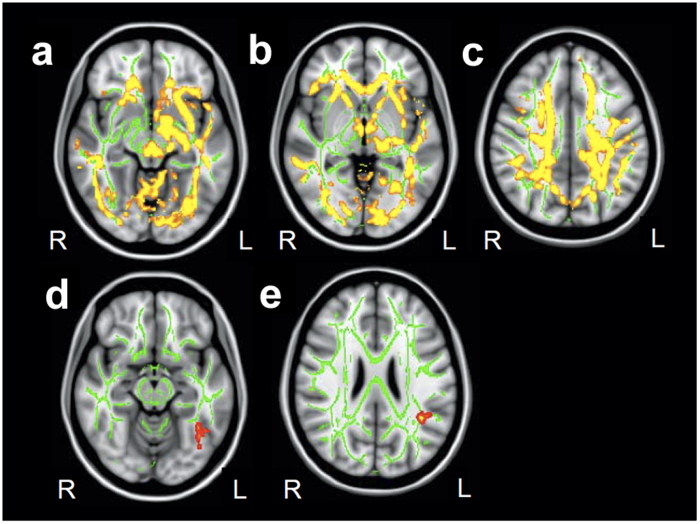
Diffusion tensor imaging analysis for patients with a high cerebrospinal (CSF) fibrinogen level. (**a–c**) The difference of fractional anisotropy (FA) values in patients with MDD and high fibrinogen levels compared with controls. Significant decreases of FA were detected diffusely. (**d, e**) The difference of FA values in patients with high fibrinogen levels compared with patients with normal fibrinogen levels. Significant decreases of FA were detected in the left inferior temporal region (**d**) and left superior temporal region (**e**). L, left; R, right. Green voxels, FA white matter skeleton; Red-yellow voxels, threshold-free cluster enhancement (p < 0.05, family wise error). The background image is the standard Montreal Neurological Institute (MNI152) brain template.

The brain MRI scan was examined for nine patients with a high CSF fibrinogen level, 20 patients with a normal fibrinogen level matched for age, sex, education, and depression severity, and 26 controls matched for age, sex, and education (Supplemental Table S2). There were significant decreases of FA value in the patients with a high fibrinogen level compared with controls (Fig. 3a–c). Among patients with a high fibrinogen level, there were significant decreases in the FA value in the left inferior temporal (Fig. 3d) and left superior temporal regions (Fig. 3e) compared with patients with a normal fibrinogen level. When we compared the FA value between patients with a normal fibrinogen level and controls, no significant difference was found in any region. In T2-weighted images, no obvious periventricular or deep white-matter hyperintensity was detected in 8 of 9 patients with a high fibrinogen level (grade 0). Only one patient with a high fibrinogen level (69 years old) had a modest hyperintensity in periventricular area (grade 2) but not in deep white matter (grade 0). We also evaluated the gray matter volume differences by performing a DARTEL analysis. However, no significant difference was detected between patients with a high fibrinogen level and patients with a normal fibrinogen level, or between patients with a high fibrinogen level and controls.

## Discussion

In the present study, we found that a subpopulation of patients with MDD had high CSF fibrinogen levels compared with controls. We also found that patients with MDD with a high fibrinogen level had white matter tract abnormalities.

The correlation analyses between the results of SOMAscan and sandwich ELISA indicated that SL000022 accurately reflects fibrinogen levels. Several studies have reported an association between elevated plasma fibrinogen levels and depression^12^,^13^,^14^, although conflicting negative results have also been reported^17^,^18^,^19^. A recent population-based study of 73,367 individuals provided strong evidence supporting the association^15^,^16^. A longitudinal study showed that elevated plasma fibrinogen levels are a risk factor for depressive symptoms in school teachers^20^. In the present study, however, there was no significant increase in mean plasma fibrinogen level, which suggests that if any effect size of plasma fibrinogen exists, it is small. However, we found that a portion of patients with MDD had an abnormally high fibrinogen level in the CSF and that mean CSF fibrinogen levels were significantly increased in patients compared with controls. To our knowledge, this is the first study to show increased CSF fibrinogen levels in patients with MDD.

Fibrinogen is the major coagulation protein in blood, and increased plasma fibrinogen levels are associated with a hypercoagulable state^21^,^22^. The observed increase in CSF fibrinogen in patients with MDD might be independent of such a systemic hypercoagulable state, because there was no correlation between CSF fibrinogen and plasma fibrinogen levels in our sample. There remains the possibility that the increased CSF fibrinogen reflects the local hypercoagulable state in the brain, which might underlie “vascular depression”^23^. However, in our MRI analyses, no leukoariosis was found in eight of nine patients with a high fibrinogen level, suggesting that CSF fibrinogen increases were not ascribed to cerebral thrombosis.

Rather, considering that fibrinogen is mainly produced in the liver and fibrinogen levels in plasma are ~1000 times higher than levels in CSF, it is feasible that high CSF fibrinogen levels are caused by leakage from the blood. In line with this hypothesis, the total protein concentration in the CSF showed a highly significant increase in patients with a high fibrinogen level compared with patients with a normal fibrinogen level. The leakage was not ascribed to the contamination while the lumbar puncture because cell count did not differ from control CSFs. Elevated CSF fibrinogen has been reported in inflammatory brain diseases such as bacterial or viral meningitis and Guillain-Barré syndrome^24^,^25^. CSF fibrinogen levels observed in these illnesses (2–8 μg/mL) are similar to levels in patients with MDD with a high fibrinogen level in the present study. The increased CSF fibrinogen in our patients could represent a trace of blood-brain barrier disruption induced by neuroinflammation, which is in accordance with the mild inflammation hypothesis in the etiology of MDD^26^,^27^. Elevated levels of cytokines have been reported not only in plasma but in the CSF of patients with MDD^11^,^27^. Further studies are needed to examine the possible relationship between fibrinogen and other inflammatory markers.

It is unlikely that the high fibrinogen levels in patients with MDD were due to medication, because at least two drug-free patients had an abnormally high fibrinogen level; in addition, there was no correlation between antidepressant dose and CSF fibrinogen levels.

Injection of fibrinogen into mice cortices induces microglial responses and the development of axonal damage^28^. Therefore, we hypothesized that the increased fibrinogen in the CSF may affect white matter fibers. The DTI allows white matter tracts to be imaged in vivo^29^ based on FA, a measure of the directionality of diffusion^30^. Degeneration of white matter tracts would be expected to result in a reduction in FA, owing to a loss of directionality of diffusion as a result of the loss of myelin and axonal membranes^31^. We found that patients with a high fibrinogen level showed lower FA values diffusively, especially in the left superior longitudinal fasciculus (SLF) and inferior temporal regions. Previous DTI studies on MDD, including ours^32^, showed a variety of abnormalities in white matter fibers including the SLF^33^,^34^ and left sagittal stratum to posterior thalamic radiation^33^. A meta-analysis of these DTI studies showed decreased white matter FA values in the left SLF^35^. These previous results are consistent with our current results.

The observation that only a part of patients had increased CSF fibrinogen might be because the increases occurred in a subgroup of the patients or the increases occurred transiently in the patients. However, the latter possibility is not in line with above mentioned structural change in the brain, which is unlikely to ascribe to transient increase of fibrinogen. Future longitudinal or follow up measurement of CSF fibrinogen should bring to resolve this debate.

In the present study, subjects were not fasted prior to lumbar punctures. In addition, sampling time was not constant and most patients were medicated. Thus, the CSF sampling was done in the “real world setting” and we could not exclude the possibility that secondary effects of MDD, such as changes in the diet or circadian rhythms, might affect CSF fibrinogen levels.

In conclusion, we identified, for the first time, a subpopulation of patients with MDD that showed increased CSF fibrinogen levels and white matter tract degeneration; these patients may represent a subtype of MDD. The increased fibrinogen levels might have caused axonal damage and subsequent depressive disorder. Future clinical and basic research studies are warranted to elucidate the mechanisms underlying the elevated CSF fibrinogen in a portion of patients with MDD and to develop a new treatment option.

## Methods

### Participants

Patients were recruited at the National Center of Neurology and Psychiatry Hospital, Tokyo, Japan, and through our website announcement. Control subjects were recruited from the community through advertisements in a free local magazine and our website announcement. All participants underwent a structured interview using the Mini-International Neuropsychiatric Interview (M.I.N.I), Japanese version^36^,^37^, administered by trained psychologists or psychiatrists. For participants with MDD, a consensus diagnosis was made according to the DSM-IV criteria^38^ on the basis of the M.I.N.I, additional unstructured interviews, and information from medical records. Participants were excluded if they had a history of central nervous system disease, severe head injury, or substance abuse. Depression severity was assessed by the Japanese version of GRID-Hamilton Depression Rating Scale (HAM-D)^39^. Antidepressants doses were converted to imipramine-equivalent doses^40^. Clinical data were managed by a database using FileMaker server (FileMaker Inc., Santa Clara, CA). This study was conducted in accordance with the Declaration of Helsinki and approved by the ethics committee of the National Center of Neurology and Psychiatry, Japan. Written informed consent was obtained from all participants.

### Lumbar puncture and venipuncture

CSF samples were obtained by lumbar puncture between 1000 h and 1600 h. After neurologic examinations, each participant received local anesthesia followed by a lumbar puncture at L3–4 or L4–5 using an atraumatic pencil point needle (Uniever 22G, 75 mm, Unisis Corp, Tokyo, Japan). The initial 2 mL of CSF was used for laboratory tests, including number of cells, total protein, and glucose. Then 8 mL of CSF was collected in a low protein adsorption tube (PROTEOSAVE SS 15 mL Conicaltube, Sumitomo Bakelite Co., Japan) and immediately chilled on ice. The CSF was centrifuged (4000 g x 10 min, 4 °C) and the supernatant was dispensed into 0.5 mL aliquots in low-protein adsorption tubes (PROTEOSAVE SS 1.5 mL Slimtube, Sumitomo Bakelite Co.) and stored in a deep freezer (−80 °C) until use. Simultaneously, blood samples were collected by venipuncture and plasma samples were obtained by centrifugation.

### SOMAscan

Workers at SomaLogic Inc. (Boulder, CO) who were blind to the diagnosis, performed the proteomic assessments as described elsewhere^41^,^42^. The assay consists of equilibrium binding of fluorophore-tagged SOMAmers and proteins in CSF and automated partitioning steps to capture only the SOMAmers that are in complexes with their cognate proteins. The assay transforms the measurement of proteins into the measurement of the corresponding SOMAmers (DNA) via hybridization to an antisense probe array. Protein concentrations were reported in relative fluorescence units (RFU).

### ELISA

Fibrinogen Human ELISA Kits (ab108841) were purchased from Abcam (Cambridge, MA). Samples were diluted to 1:50 for the CSF and 1:10000 for the plasma using the diluent attached to the kit. An ELISA kit for Fibrin D-dimer (#RK023A) was purchased from Hyphen BioMed (Neuville-sur-Oise, France), and CSF samples were diluted to 1:4 using the sample diluent attached to the kit. An ELISA kit for fibrinogen gamma (SEC477Hu) was purchased from Uscn Life Science Inc. (Wuhan, China) and CSF samples were diluted to 1:10 using phosphate buffered saline.

### MRI data acquisition and processing

MRI was performed on a Magnetom Symphony 1.5-tesla (Siemens, Erlangen, Germany). High spatial resolution, 3-dimensional (3D) T1-weighted images, diffusion tensor imaging (DTI), conventional T2-weighted images, and fluid attenuation inversion recovery images of the brain were obtained for the morphometric study. Detailed information on MRI parameters are described elsewhere^43^. On conventional MRI, no abnormal findings were detected in the brain of any subject. Each individual 3D-T1 image was normalized with the diffeomorphic anatomical registration using exponentiated lie (DARTEL) registration method^44^ using Statistical Parametric Mapping 8 (Wellcome Trust, University College London, UK) software. DTI metrics were calculated by using Tract-Based Spatial Statistics (TBSS) analysis^45^. A fractional anisotropy (FA) threshold of 0.20 or higher was set to exclude peripheral tracts. Evaluation of the leukoaraiosis was performed on T2-weighted images using Fazekas Scale by a single person (M.O.) in a blinded manner^46^.

### Statistical analysis

The SOMAscan results for 3 fibrinogen-related proteins were first analyzed using Microsoft Excel (Excel 2010, Microsoft Corporation, Redmond, WA). The cut off value for each protein was determined as the 99 percentile of 30 control subjects using the “PERCENTILE” function. Further analyses were performed with the SAS program (The SAS Enterprise guide 5.1, SAS Institute Inc., Cary, NC). Analysis of covariance (ANCOVA, type III sum of squares) was used to identify the effects of diagnosis on quantitative values (such as fibrinogen levels) or the effect of fibrinogen levels (high/normal) on quantitative values (such as HAM-D scores). The relationships among protein levels (measured by SOMAscan or ELISA) were analyzed by Pearson’s correlation test. The increases in the number of patients with high fibrinogen levels were assessed by one-sided Fisher’s exact test. Odds ratio (OR) and 95% confidence intervals (CI) were also obtained by the SAS program. Z scores were calculated using the mean and standard deviation of the control group in each sample set using the following formula; (value - mean)/(standard deviation).

We prepared three MDD and age-, sex- matched control sample sets. SOMAscan was performed on a “first sample”, consisting of 30 MDD patients and 30 controls (Table 2). CSF fibrinogen levels were then analyzed by ELISA using a “second sample” consisting of 36 MDD patients and 30 controls (Table 2). Plasma fibrinogen was examined on a “plasma sample” consisting of 26 patients and 27 controls (Supplemental Table S3). There were no significant differences in age, sex, sampling time, cell number, or total protein levels between the diagnostic groups for the two CSF sample sets. There was no overlap of subjects between the first and second samples, while 70 subjects overlapped between the CSF second and the blood fibrinogen samples.

## Additional Information

**How to cite this article**: Hattori, K. *et al.* Increased cerebrospinal fluid fibrinogen in major depressive disorder. *Sci. Rep.* **5**, 11412; doi: 10.1038/srep11412 (2015).

## Supplementary Material

Supplementary Information

## Figures and Tables

**Table 1:** Comparison between patients with MDD with normal and high CSF fibrinogen levels.

	**Normal fibrinogen**	**High fibrinogen**	**p value**
N	51	15	
Male/Female	23/28	8/7	n.s.
Age	41.4 ± 9.5 (22–63)	51.7 ± 15.2 (37–80)	0.0008
Elapsed time from 10:00(min)	207.8 ± 97.2 (0–390)	190.9 ± 106.4 (0–360)	n.s.
HAM-D 17	13.2 ± 8.1 (0–31)	20.3 ± 7.8 (8–39)	n.s.
IMI-equivalent (mg/day)	140.6 ± 145.0 (0-525)	146.2 ± 127.1 (0–400)	n.s.
CSF WCC (/mL)	3.8 ± 2.5 (0–11)	3.1 ± 1.8 (0–7)	n.s.
CSF Protein (mg/dL)	38.4 ± 10.6 (21-72)	66.4 ± 19.2 (38–107)	1.9 × 10^−8^

MDD, major depressive disorder; CSF, cerebrospinal fluid; HAM-D 17, 17-item version of Hamilton Depression Rating Scale; IMI, imipramine; WCC, white cell count. Values are mean ± standard deviation. Values in the parentheses indicate ranges.

**Table 2:** Demographic and clinical characteristics of the cerebrospinal fluid samples.

	**First sample**			**Second sample**		
	**Control**	**Control**	**MDD**	**Control**	**Control**	**MDD**
N	30	30		30	36	
Male/Female	15/15	15/15	n.s.	15/15	16/20	n.s.
Drug free	—	10		—	4	
Age	41.9 ± 14.3 (20–72)	44.2 ± 12.3 (23–80)	n.s.	40.7 ± 9.9 (22–58)	43.3 ± 11.5 (22–76)	n.s.
Elapsed time from 10:00 (min)	160.5 ± 124.4 (0–360)	185.8 ± 104.3 (0–360)	n.s.	202.9 ± 110.2 (0–355)	218.7 ± 92.8 (0–390)	n.s.
HAM-D 17	N/A	16.5 ± 8.0 (0–31)		N/A	13.4 ± 8.8 (1–39)	
IMI-equivalent (mg/day)	N/A	142 ± 135 (0–400)		N/A	136.5 ± 147.9 (0–525)	
CSF WCC (/mL)	4.9 ± 3.7 (0–14)	4.4 ± 2.7 (0–11)	n.s.	3.5 ± 2.5 (0–10)	3.1 ± 2.0 (0–8)	n.s.
CSF protein (mg/dL)	38.0 ± 8.2 (24–59)	43.9 ± 18.2 (21–107)	n.s.	35.2 ± 8.7 (20–60)	43.3 ± 18.1 (1–97)	n.s.

MDD, major depressive disorder; HAM-D 17, 17-item version of Hamilton Depression Rating Scale; IMI, imipramine; WCC, white cell count. Values are mean ± standard deviation. Values in the parentheses indicate ranges.
