# Supplementary material for: Increased cerebrospinal fluid fibrinogen in major depressive disorder
Source: Sci Rep. 2015 Jun 17;5:11412. doi: 10.1038/srep11412 (PMC4469953; doi:10.1038/srep11412)
Supplement: Supplementary Information [file srep11412-s1.pdf]

Increased cerebrospinal fluid fibrinogen in major depressive disorder

Kotaro Hattori[1,2,*], Miho Ota[1], Daimei Sasayama[1], Sumiko Yoshida[3], Ryo Matsumura[2], Tomoko Miyakawa[2], Yuuki Yokota[2], Shinobu Yamaguchi[2], Takamasa Noda[3], Toshiya Teraishi[1], Hiroaki Hori[1], Teruhiko Higuchi[4], Shinichi Kohsaka[5], Yu-ichi Goto[2], Hiroshi Kunugi[1]

[1]Department of Mental Disorder Research, National Institute of Neuroscience, National Center of Neurology and Psychiatry, Tokyo 187-8502, Japan

[2]Translational Medical Center, National Center of Neurology and Psychiatry, Tokyo 187-8551, Japan

[3]National Center of Neurology and Psychiatry Hospital, Tokyo 187-8551, Japan

[4]National Center of Neurology and Psychiatry, Tokyo 187-8551, Japan

[5]National Institute of Neuroscience, National Center of Neurology and Psychiatry, Tokyo 187-8502, Japan

Figure S1. Correlation between SL000022 and SL000424

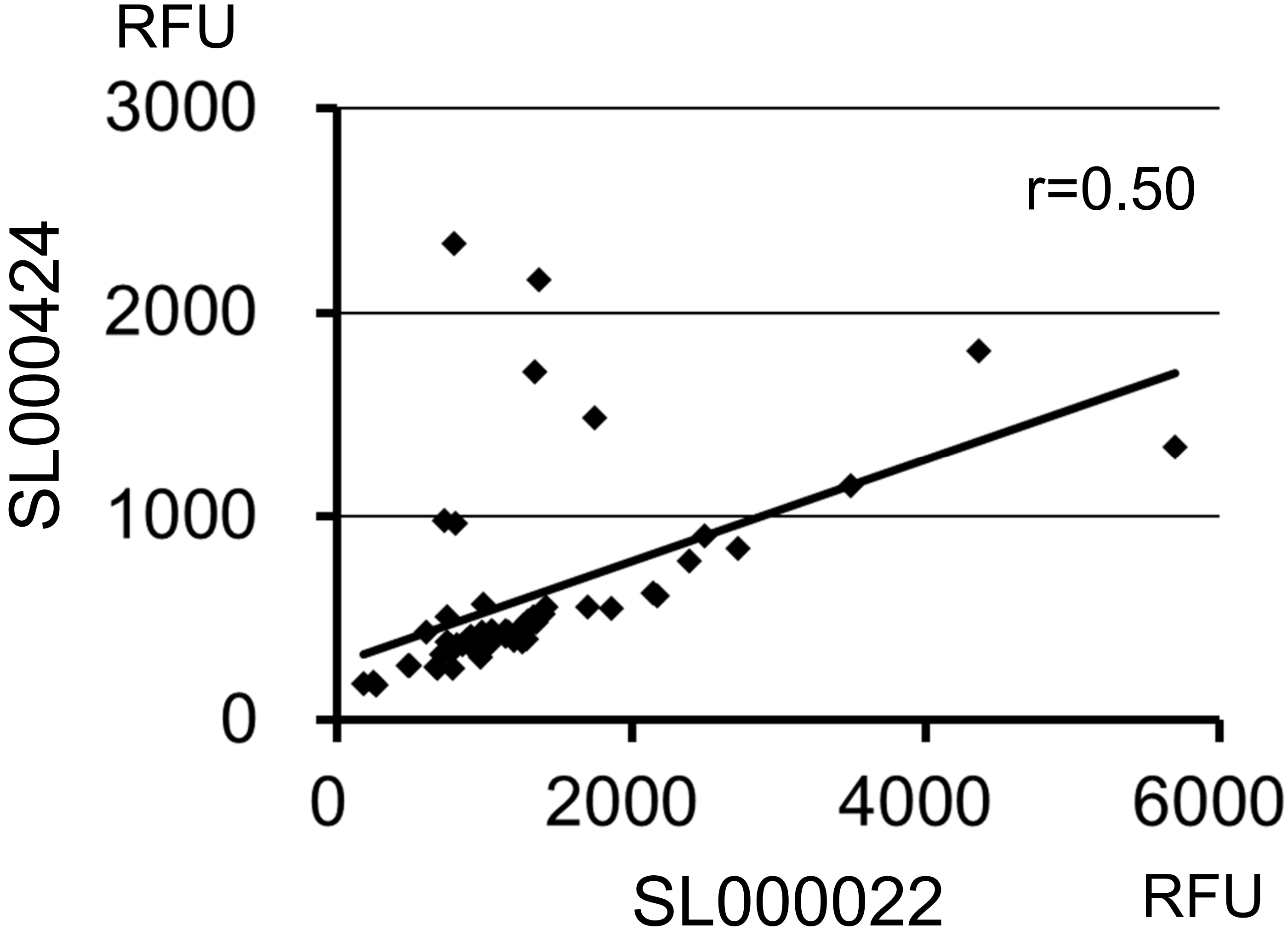

Figure S2. Correlation between SL000022 and SL003341

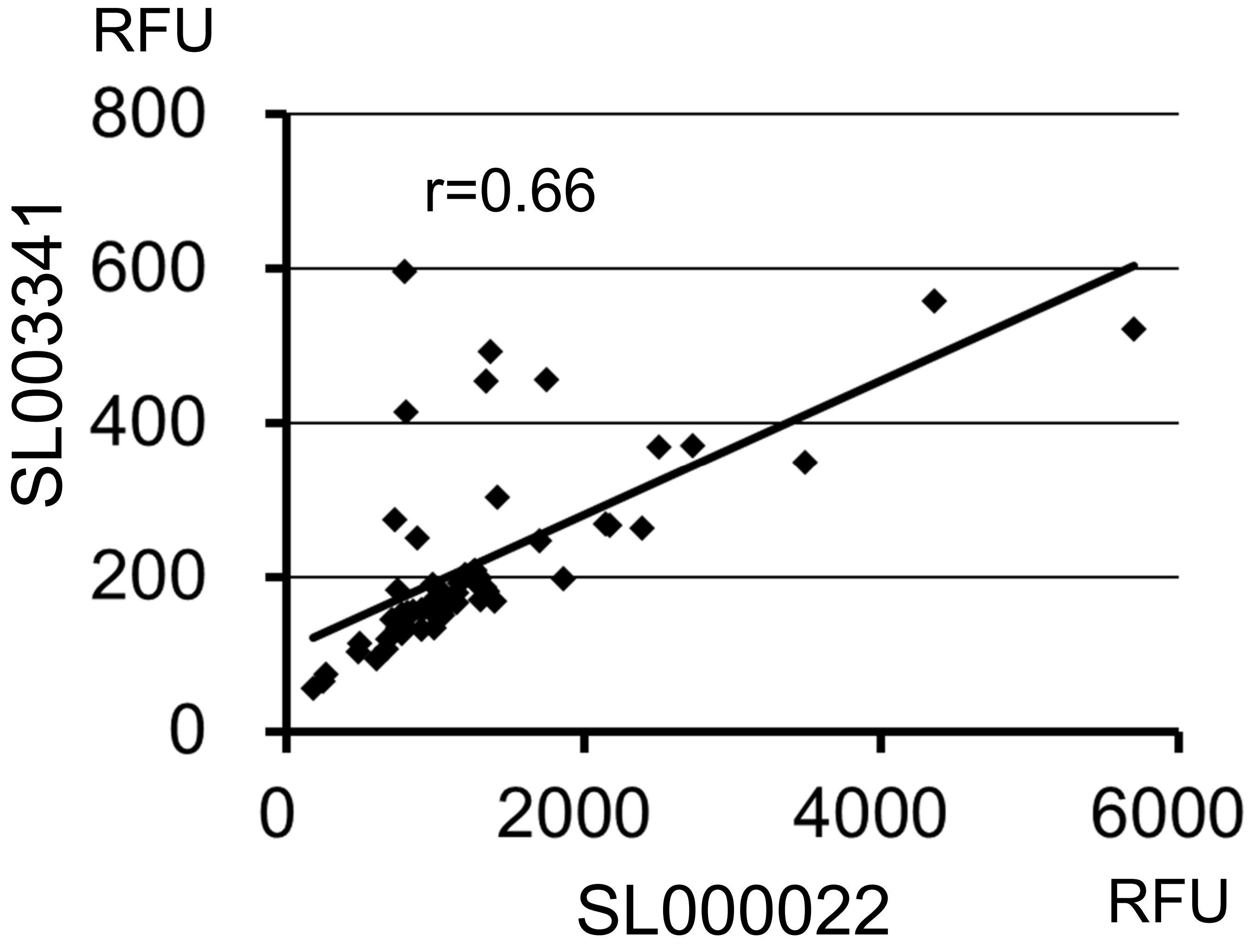

Figure S3. Correlation between SL000424 and SL003341

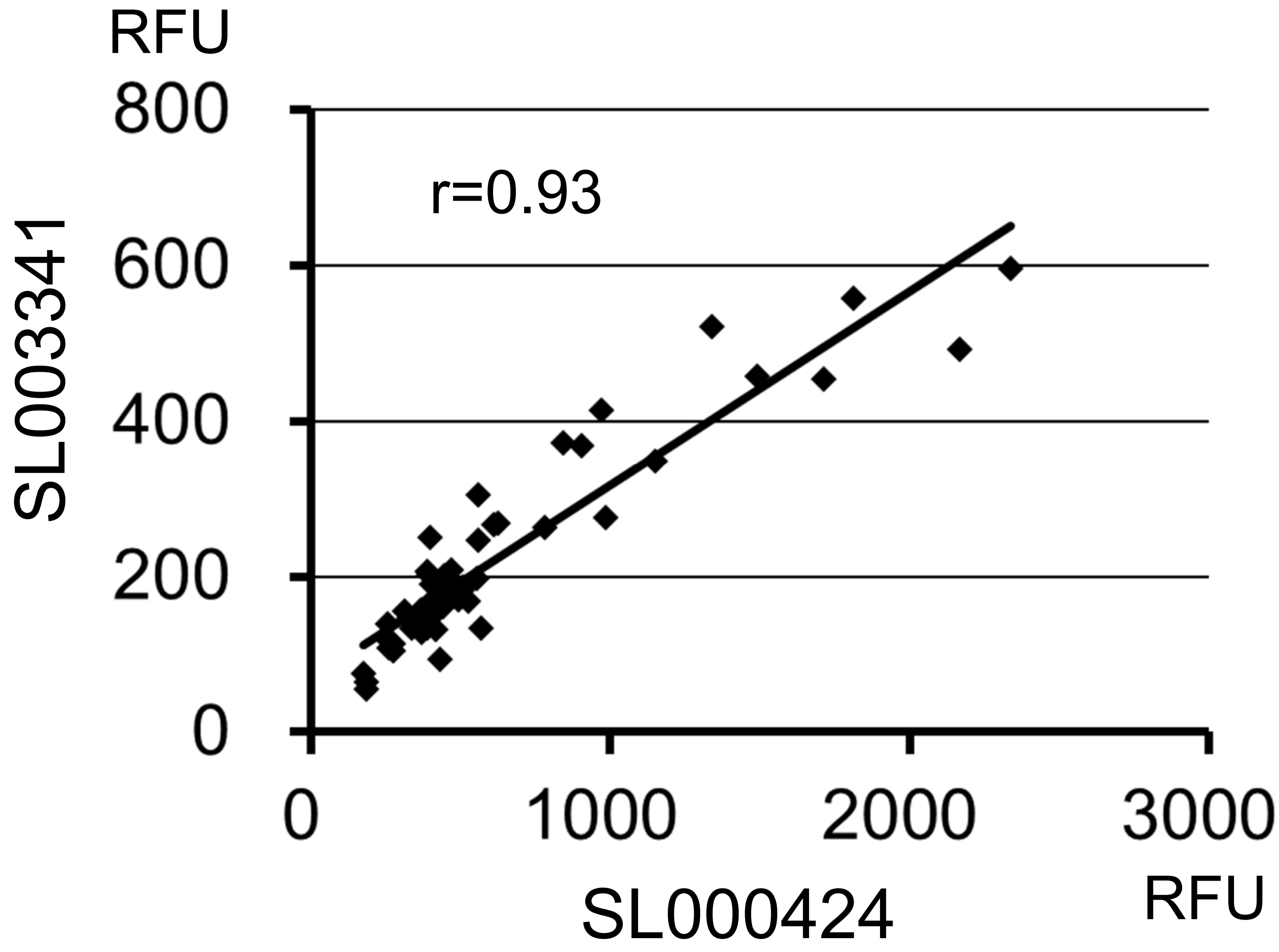

Figure S4. Correlation between fibrinogen γ-chain (ELISA) levels and SL000022

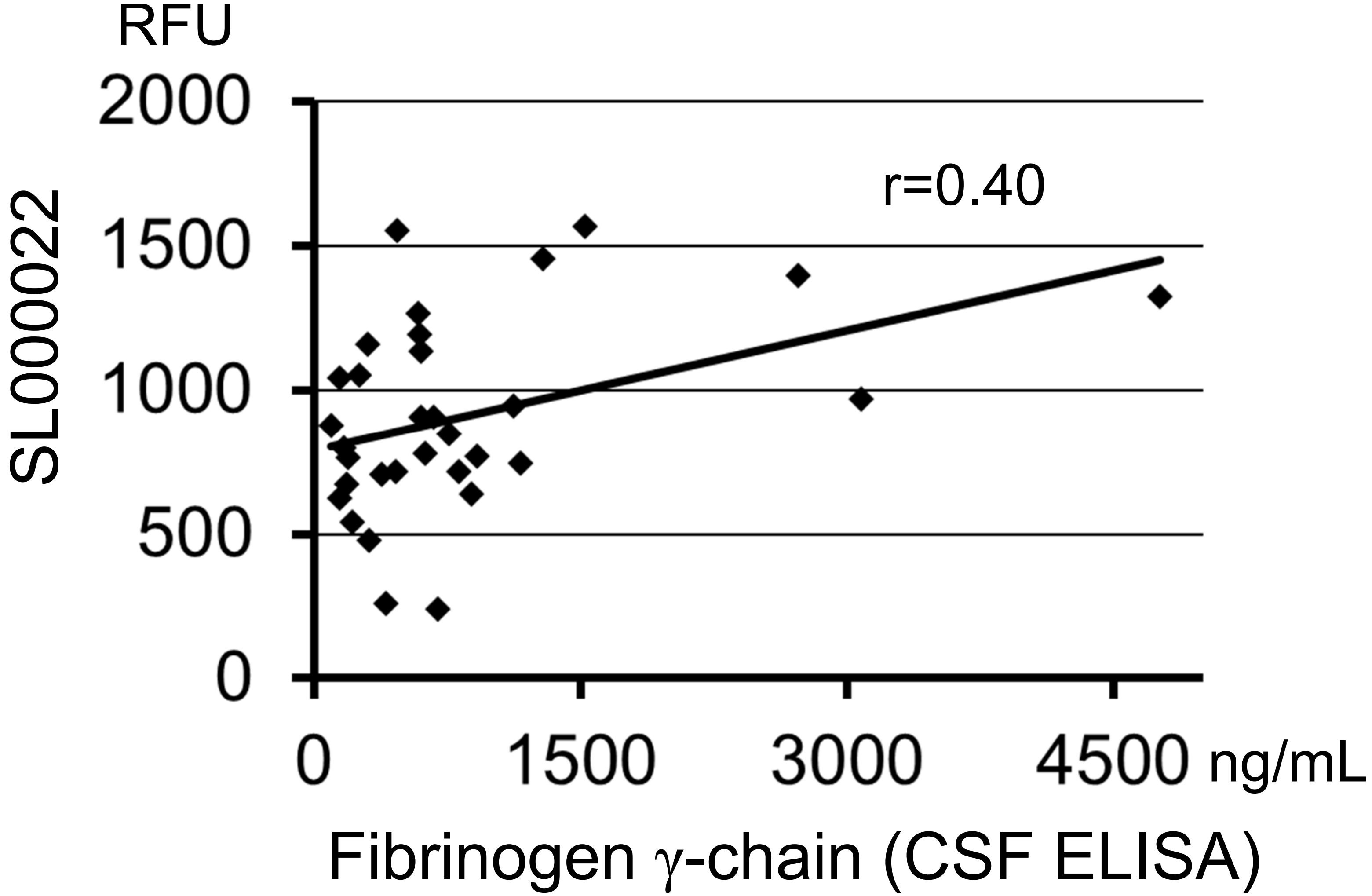

Figure S5. Ages of patients with "normal " and "high" CSF fibrinogen level

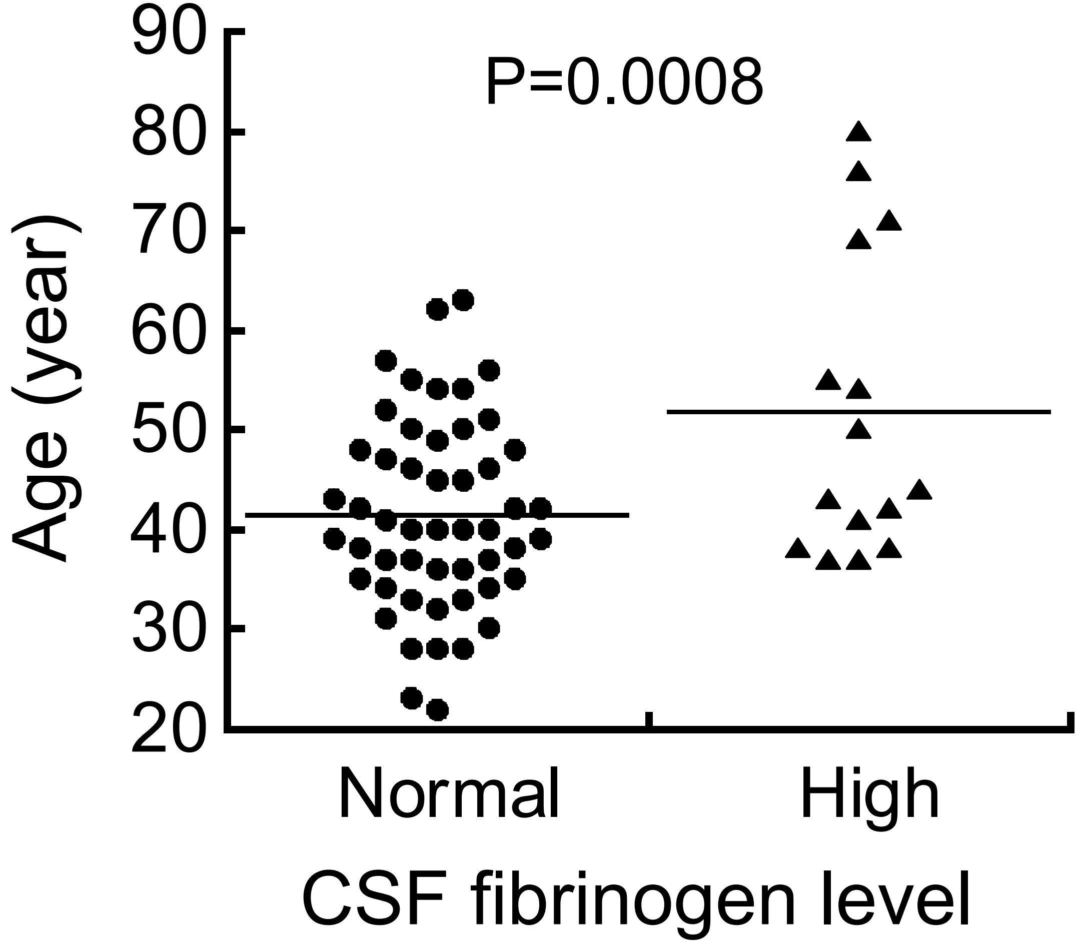

Figure S6. HAM-D17 scores of patients with "normal" and "high" CSF fibrinogen level

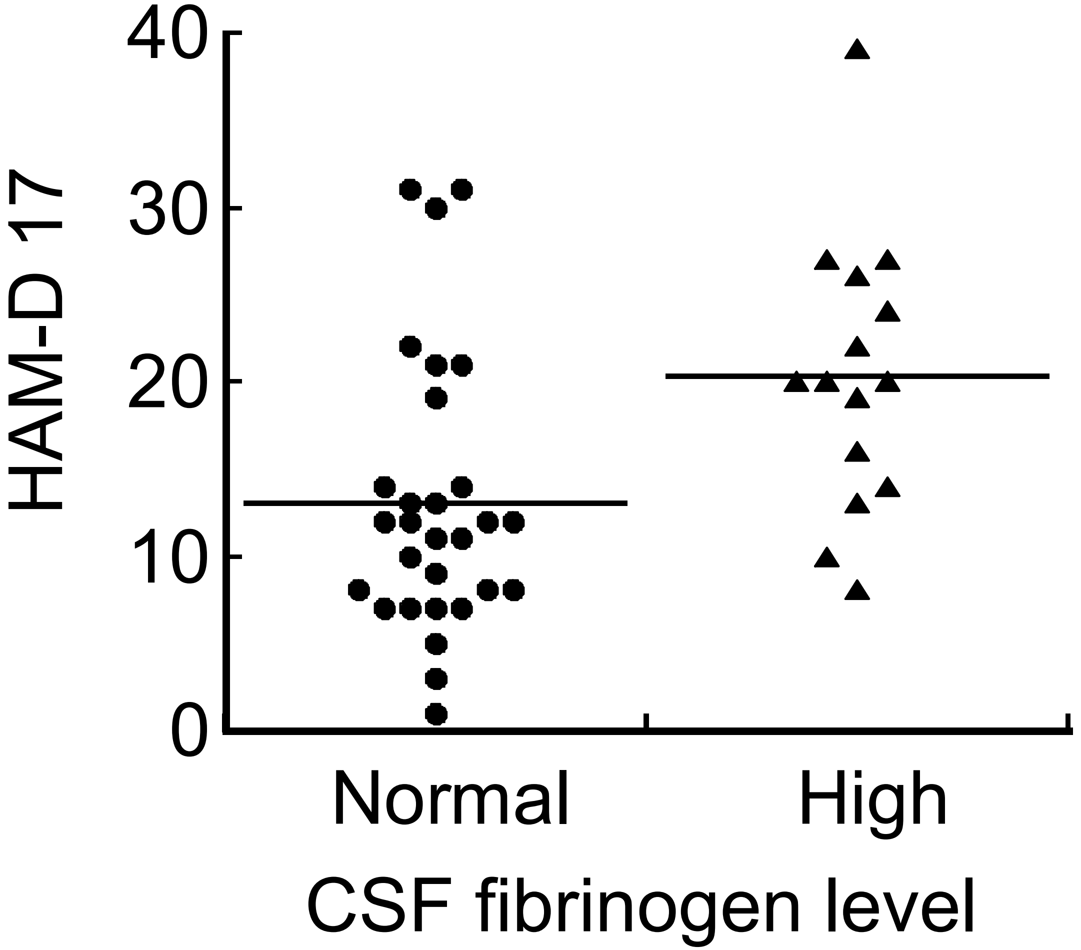

Fig.S7 Correlation between CSF and plasma fibrinogen levels

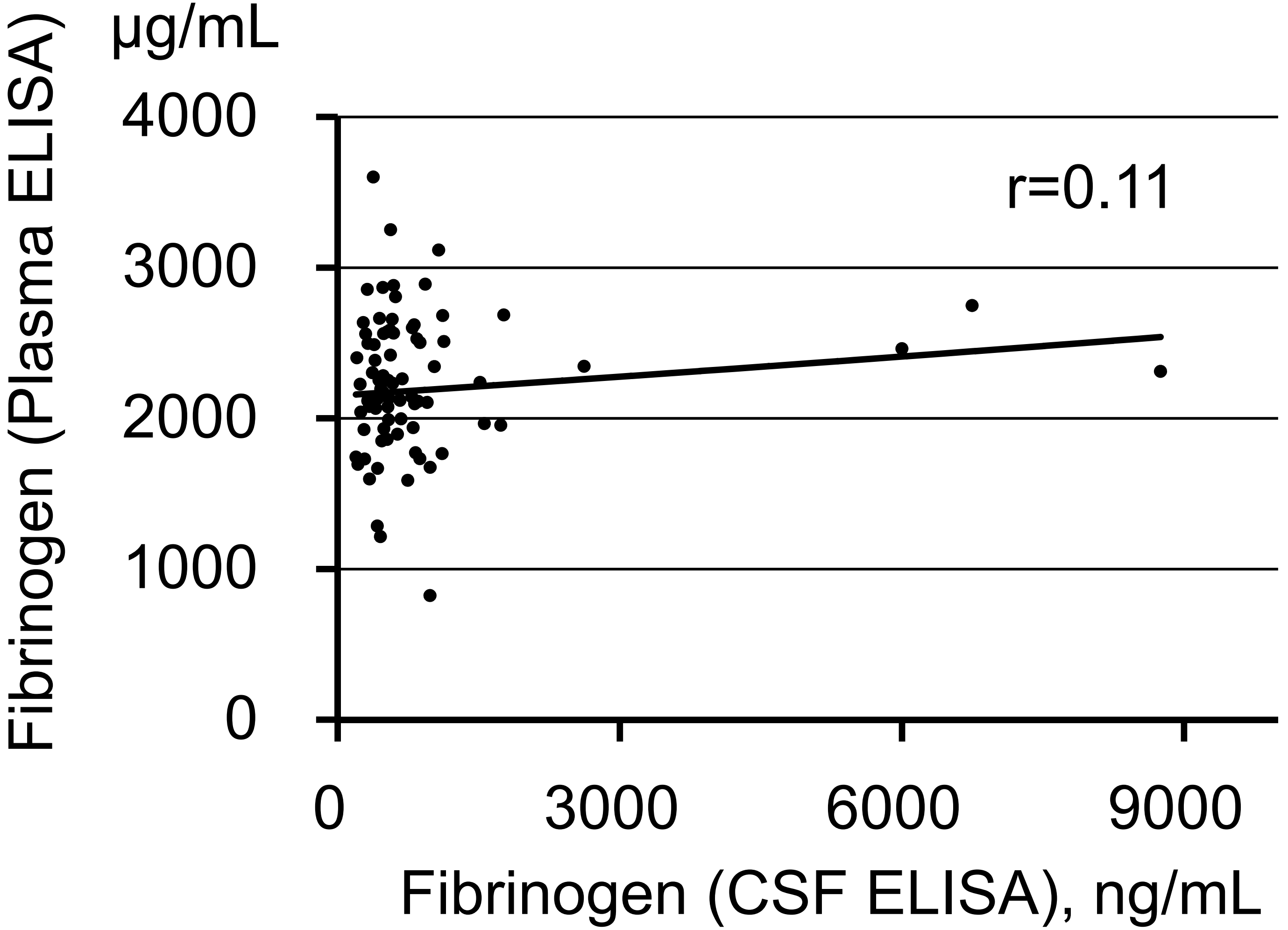

Supplemental figure legends

Figure S1. Correlation between SL000022 and SL000424. RFU, relative fluorescent unit; r, Pearson's correlation coefficient.

Figure S2. Correlation between SL000022 and SL003341. RFU, relative fluorescent unit; r, Pearson's correlation coefficient.

Figure S3. Correlation between SL000424 and SL003341. RFU, relative fluorescent unit; r, Pearson's correlation coefficient.

Figure S4. Correlation between fibrinogen γ-chain (CSF ELISA) levels and SL000022. RFU, relative fluorescent unit; r, Pearson's correlation coefficient; CSF, cerebrospinal fluid; ELISA, enzyme-linked immunosorbent assay.

Figure S5. Ages of patients with "normal" and "high" CSF fibrinogen levels. Plain lines, average in each group; Normal, patients with major depressive disorder and normal fibrinogen levels; High, patients with major depressive disorder and high fibrinogen levels.

Figure S6. HAM-D17 scores of patients with "normal" and "high" CSF fibrinogen levels. Plain lines, average in each group; Normal, patients with major depressive disorder and normal fibrinogen levels; High, patients with major depressive disorder and high fibrinogen levels.

Figure S7. Correlation between CSF and plasma fibrinogen levels. r, Pearson's correlation coefficient; CSF, cerebrospinal fluid; ELISA, enzyme-linked immunosorbent assay.

Supplemental Table S1. Pearson's correlation coefficient between SomaScan and ELISA (CSF)

	ELISA (CSF)		
SOMAscan	Fibrinogen	Fibrinogen γ-chain	D-dimer
SL000022	0.77**	0.40*	0.27
SL000424	0.31	0.31	0.23
SL003341	0.28	0.12	0.08

Values are Pearson's correlation coefficient (r); *p<0.05; **p<0.001.

Supplemental Table S2. Demographic and clinical characteristics of the MRI scan sample

	MDD with high-fibrinogen	MDD with normal fibrinogen	Control	p value
Male / Female	5 / 4	10/10	12 / 14	n.s.
Age (years)	50.7 ± 12.7	43.9 ± 8.7	44.8 ± 14.2	n.s.
Education (years)	15.7 ± 5.0	14.5 ± 2.2	15.1 ± 2.0	n.s.
IMI-equivalent (mg/day)	172.9 ± 185.7	116.8 ± 116.0		n.s.
HAM-D 17	22.7 ± 9.0	18.9 ± 7.3		n.s.

MRI, magnetic resonance imaging; MDD, major depressive disorder; HAM-D 17, 17-item version of Hamilton Depression Rating Scale; IMI, imipramine. Values are mean ± standard deviation.

Supplemental Table S3. Demographic and clinical characteristics of the "plasma sample"

	Control	MDD	p value
N	27	26	
Male/Female	14/13	13/13	n.s.
Drug free		4	
Age	41.6 ± 9.1 (25-58)	41.4 ± 7.3 (28-56)	n.s.
Elapsed time from 10:00(min)	167.1 ± 118.6 (0-345)	219.0 ± 75.6 (0-360)	n.s.
HAM-D 17	N/A	11.0 ± 6.8 (1-30)	
IMI-equivalent (mg/day)	N/A	118.4 ± 135.9 (0-375)	

MDD, major depressive disorder; HAM-D 17, 17-item version of Hamilton Depression Rating Scale; IMI, imipramine. Values are mean ± standard deviation. Values in the parentheses indicate ranges.
